# Barriers and facilitators to implementing an online psychological intervention for adolescent and young adult cancer survivors in a community setting

**DOI:** 10.1007/s00520-026-10867-9

**Published:** 2026-06-08

**Authors:** Elin Irestorm, Claire E. Wakefield, Rachel Houweling, Kate Hetherington, Brittany C. McGill, Fiona E. J. McDonald, Richard J. Cohn, Ursula M. Sansom-Daly

**Affiliations:** 1 Faculty of Medicine, Department of Paediatrics, Lund University, Lund, Sweden; 2 School of Clinical Medicine, Discipline of Paediatrics and Child Health, Behavioural Sciences Unit, Randwick Clinical Campus, UNSW Medicine and Health, UNSW, Randwick, NSW Australia; 3 Division of Quality of Life and Pediatric Palliative Care, Department of Pediatrics, Stanford University and Stanford Medicine Children’s Health, Palo Alto, CA USA; 4 Kids Cancer Centre, Sydney Children’s Hospital, Randwick, Australia; 5 Canteen, Newtown, Australia; 6 Faculty of Medicine and Health, The University of Sydney, Sydney, NSW Australia; 7 Sydney Youth Cancer Service, Nelune Comprehensive Cancer Centre, Prince of Wales Hospital, Randwick, Australia

**Keywords:** Cancer survivors, Psycho-oncology, Cognitive behavioural therapy, Implementation science

## Abstract

**Purpose:**

To respond to the psychological needs of adolescent and young adult (AYA) cancer survivors, we developed and evaluated a theoretically grounded online intervention to teach adaptive coping skills to promote resilience in early survivorship: ‘Recapture Life’. This qualitative study aimed to evaluate the process of implementing Recapture Life into community organisations, after having been initially developed in an academic setting.

**Methods:**

The study duration was 2017–2022. Eighteen community staff members were interviewed before the implementation (T0) and nine of them after the implementation (T1). A qualitative data process evaluation using the Proctor model for implementation was completed. Interviews were thematically analysed and mapped to four key domains: acceptability, adoption, appropriateness, and sustainability.

**Results:**

Major facilitators included the training provided to staff before implementation, support from the community organisation, and the culture within the organisation, as well as the communication between the university/hospital and the community organisation/s. Major barriers included staff turnover and recruitment of participants. Most, but not all, of the potential barriers and facilitators mentioned at T0 were confirmed at T1. The COVID-19 pandemic was considered both a barrier and a facilitator. Additionally, this category did not fit into the Proctor framework, as it cut across all outcomes.

**Conclusion:**

We identified several processes critical to supporting the success of an AYA cancer intervention delivery. This study highlights the potential for psychological programs to be delivered online by skilled and well-trained psychosocial staff in community and not-for-profit settings.

Trial registration

The study was registered in the Australian New Zealand Clinical Trials Registry (ACTRN126240013995830) November 26th, 2024 (retrospectively registered).

## Background

Cancer survivors often face difficulties in resuming normal activities, including work and social interactions, after treatment completion [1, 2]. A recent large-scale meta-analysis of epidemiological studies showed that the median onset of mental health disorders is during late adolescence [3]. For adolescents and young adults (AYAs) diagnosed with cancer, the intersection of developmental vulnerabilities with cancer-related stressors means that their distress can be more complex to manage than that of other age groups [4–6]. The broadest international definition of AYAs are ages 13–39 years [7]. To respond to the psychological needs of AYA cancer survivors, we developed and evaluated an online psychological group intervention to teach adaptive coping skills and promote resilience in early survivorship called ‘Recapture Life’ [8]. The intervention was developed by an expert team (including people with lived experience) using gold-standard guidelines [9, 10]. Recapture Life is preventive and targets psychological mechanisms that maintain post-cancer distress using cognitive-behavioural therapy (CBT) strategies. While systematic review data has shown that CBT is effective regardless of therapy delivery modality and across all treatment phases except amongst those newly diagnosed, almost all extant studies involved older participants [11]. Previous studies have demonstrated the Recapture Life programme’s clinical safety [12] and its ability to support positive therapeutic processes in the online setting, from both AYA and group-facilitator perspectives [13, 14]. We also demonstrated its acceptability and feasibility [15], participants’ acquisition and use of adaptive coping strategies learned through CBT skills [16], and increased peer-support and decreased cancer-related problems [17].

Despite these promising data, the lack of mental health professionals in Australian paediatric/AYA oncology poses a barrier to implementing this model of care more widely in hospital settings. Furthermore, there is considerable variation between hospitals regarding what psychological services are available and for how long post-treatment AYA cancer survivors can access these services [18]. The Recapture Life program was initially delivered by hospital-based therapists, funded via a research grant in academic settings [8, 19]. However, in Australia, community organisations, external to the health system, are increasingly providing a platform for broader education around psychological concerns post-treatment, as well as supporting survivors and families through the provision of resources and interventions [18, 20]. Partnering with the community sector to enhance access to efficacious online models of care may improve AYAs’ psychosocial outcomes in survivorship as they transition away from hospital settings, whilst also reducing the burden on the health system.

Implementing a new intervention, regardless of delivery modality, requires attention to facilitators and barriers. Previous research has shown that psycho-oncology implementation challenges are multifaceted and occur across various domains, including changes in clinical care processes, clinician knowledge, and organisational culture [21]. For such multifaceted challenges, the Proctor framework provides a structured approach to assessing key implementation outcomes [22]. When identifying barriers to the successful implementation of psychosocial care in cancer, previous studies have reported more institutional barriers than those related to individual provider or patient characteristics [23]. In line with these results, a study investigating Australian health professionals’ perceptions of the feasibility and acceptability of a community-based model of psycho-oncology care revealed that most clinicians were willing to adopt the proposed changes into practice [24]. The staff delivering an intervention are uniquely positioned to provide valuable insights into the barriers and facilitators to implementing healthcare interventions in the settings in which they work [25], making them a valuable source of information when evaluating implementation processes.

### Aims

To implement Recapture Life in the community, we partnered with three Australian community-based cancer support organisations (hereafter referred to as ‘community organisations’). This study aimed to evaluate the process of implementing Recapture Life (initially developed in a university/hospital setting) into community settings, focusing on facilitators and barriers. Our research questions were (1) what potential barriers and facilitators did staff anticipate before implementation and (2) according to staff, which barriers and facilitators to implementation emerged in line with their expectations after delivering the program?

## Methods

### Design and context

In Australia, community organisations provide cancer counselling, peer connection, financial support, transport to treatment, accommodation, and legal and workplace support. We partnered with three community organisations for this study: Canteen [26], Cancer Council New South Wales (CCNSW) [27], and Country Hope [28].These were selected due to their established capabilities to deliver psychosocial support to AYAs with cancer, including via online/telehealth methods. Within this implementation, the intervention was delivered by trained staff within community organisations and offered alongside each organisation’s existing services. The program and materials were adapted to fit the groups targeted by the different organisations. For example, given CCNSW’s older age range, Recapture Life content was tailored to be relevant to older AYA survivors and renamed ‘Re-Claim Life’. The intervention format was not changed in any community or setting. Survivors received six weekly, 90-min online sessions (delivered by two staff members) and an individual booster session (delivered by 1–2 staff members) 6 weeks after the last group session. The AYAs were eligible for the intervention if they were aged 13–39 and had completed curative intent treatment.

### Procedures

**Fig. 1:**
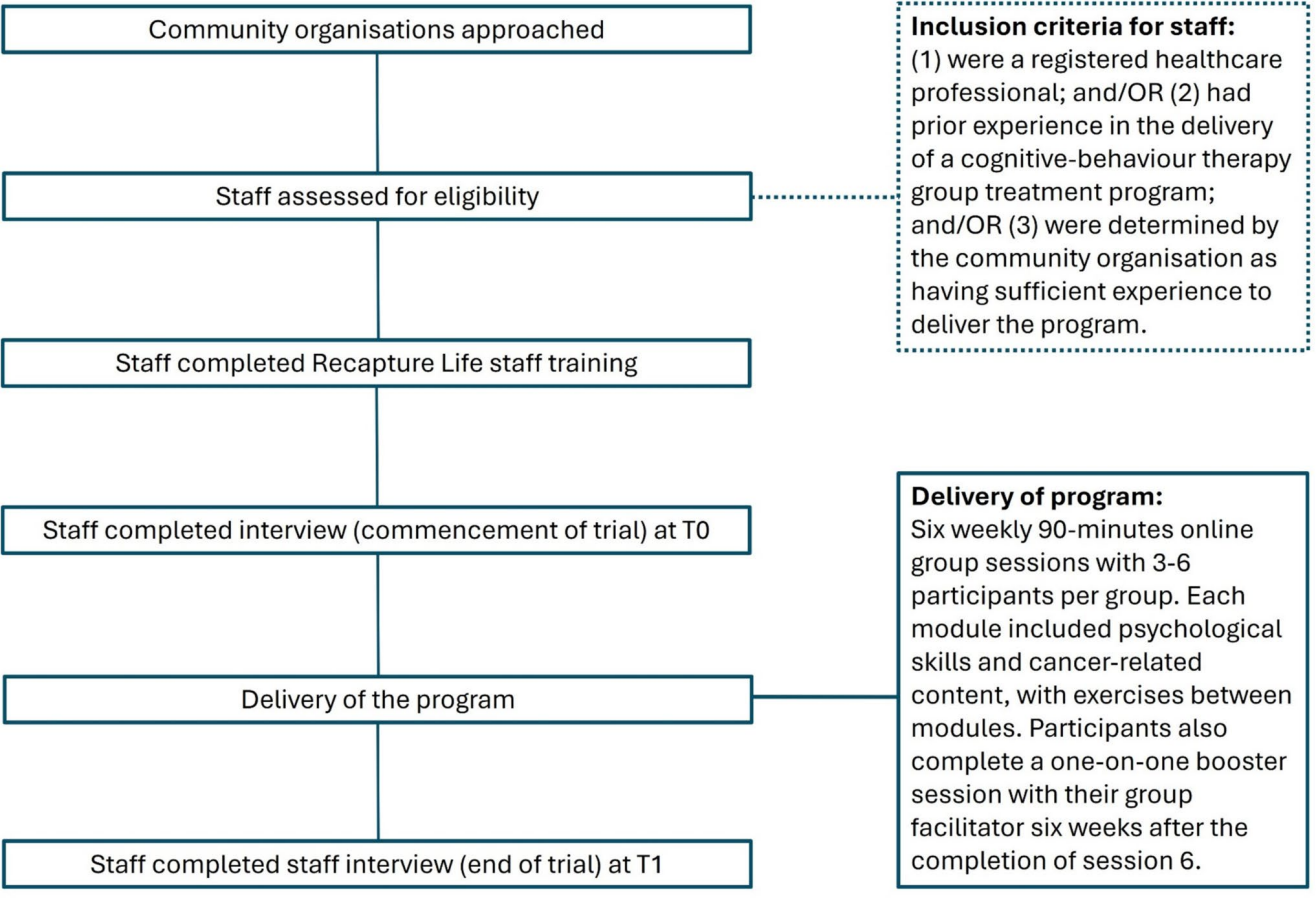
Flow of community organisation staff through the Recapture Life implementation study

After identifying how Recapture Life might fit with the community organisations’ existing services, the organisations’ informational and training needs were assessed. Alongside the Recapture Life manual, the healthcare professionals were also provided with in-person training by three of the co-authors (USD, KH, and BM). Feedback from staff was sought after each training session, and subsequent training sessions addressed identified areas of need. Once training was complete, advertising, recruitment, and intervention delivery began. During this time, the research team provided the community organisation staff with regular supervision. The flowchart for community staff is shown in Fig. 1. Table 1 depicts participant demographics.

**Table 1:** Participant demographics (*N* = 18)

Gender, *N* (%)	Female: 15 (83%) Male: 3 (17%)
Community organisation, *N* (%)	Canteen: 8 (44%) CCNSW: 9 (50%) Country Hope: 1 (6%)
Highest level of education, *N* (%)	Non-university certificate: 2 (11%) Bachelor: 3 (17%) Master: 10 (56%) PhD: 3 (17%)
Age at first interview, mean (range)	43.7 (30–59)
Years working for organisation, mean (range)	4.6 (0.5–13)
Years working in profession, mean (range)	10.5 (2–27)

### Participants

Each community organisation nominated staff to deliver Recapture Life. Staff members were eligible to deliver Recapture Life if they (1) were a registered psychosocial healthcare professional, and/or (2) had prior experience in the delivery of a CBT group treatment program, and/or (3) were determined by the community organisation as having sufficient experience to deliver the program due to the requirements/experience involved in their current psychosocial support role.

Nineteen staff members were invited to participate. One staff member declined participation, resulting in 18 staff being interviewed at T0 (95% opt-in rate). At T1, seven staff members were no longer working for the community organisation and two were on leave, resulting in nine staff participating. Most participants were female (83%), and the mean age at first interview was 43.7 years (range 30–59). The mean time working for the organisation was 4.6 years, and the mean time working in the profession was 10.5 years. Table 1 depicts participant demographics.

### Interviews

T0 interviews were conducted between October 2017 and July 2021 and T1 interviews between October 2019 and October 2022. The interviews were conducted by a trained research assistant from the Behavioural Sciences Unit, at UNSW Sydney. Staff members were interviewed at two timepoints: T0, after they had received training but before they started their first group, and T1, after they had finished their last group (of the implementation trial). Apart from demographic questions (gender, age, highest degree, time working for the organisation, and total time working in the profession), the participants were asked the same three open-ended questions at T0 and T1:

1. What will be/were the *barriers* to successfully implementing the Recapture Life program at your organisation?
2. What will be/were the *facilitators* to implementing the Recapture Life program at your organisation?
3. Do you have any other comments regarding the implementation of the Recapture Life program at your organisation?

### Data management

We audio-recorded and transcribed the interviews. The transcripts were coded using NVivo Version 15 (QSR International). We used a deductive content approach to analyse the qualitative data [29]. To guide our exploration, we adopted the Proctor conceptual framework [22], which conceptualises different outcomes key to improving implementation. Of these, four outcomes from the framework apply to semi-structured interviews: acceptability, adoption, appropriateness, and sustainability. Acceptability includes satisfaction with various aspects of the innovation, such as content, complexity, comfort, delivery, and credibility. Adoption is defined as uptake, utilisation, initial implementation, and intention to try. Appropriateness is defined as the perceived fit, relevance, compatibility, suitability, usefulness, and practicability. Sustainability is defined as maintenance, continuation, durability, incorporation, integration, institutionalisation, sustained use, and routinisation. 

### Analysis

One author (EI) coded the data and, through discussion with another co-author (USD), created subcategories to map onto the four implementation outcomes. To create subcategories, a categorisation matrix was created before coding, and the subcategories were derived within each category. A third author (RH) was engaged to assess the subcategories after the initial coding and review the framework fit. Analytic decisions, coding matrices, and feedback were tracked. The interviews from T0 and T1 were analysed separately, since T0 was conducted between the training and the delivery of the intervention, whereas T1 was conducted after the delivery. Reporting follows the COREQ guidelines, which were designed to ensure complete, transparent, and rigorous reporting of qualitative studies using interviews or focus groups [30]. Due to the smaller sample size at T1, saturation was judged relative to the stability of the themes rather than participant count. Participant quotes are presented to illustrate the categories, with quotes noted with the code P and a number (e.g., P1). Participants who were interviewed a second time retained their number from the first interview. Exemplar quotes from T0 are presented in Table 2 and T1 in Table 3.

**Table 2:** Outcomes and categories from before the implementation (T0)

T0 outcomes	Categories	Exemplar quotes
Acceptability barriers	Reach	*“It’s not a peer support group if there’s not enough people. So, I guess my experience is it’s always better to get more people to commit, so that if there is a dropout rate that you can continue, you still have a peer group to keep going. So, I think the numbers are really important. So, the recruitment’s really important.”* P9
	Progress	“*There may be other contingencies from the university perspective, that will influence the flow of the project. I suppose an example of that was with the signing of the contract, the - that was delayed.”* P11
Acceptability facilitators	Relationships	*“So we’ve got you guys as the research team and then we’ve got us delivering it and we’ve delivered a few programs of a similar nature before… so we have quite a bit of experience in running that sort of stuff and then we’re backed up by you guys as well so I think that’s a big plus.”* P2
	Structure	“*When they said, oh no, just relax on this, work around the model, work within the frame, but make sure that the frame actually is a very adaptable and flexible to the learning needs of the participants in the group. I sighed a sigh of relief.”* P12
Adoption barrier	Progress	*“Possibly I would guess you know, actually having said, the one thing that might be an issue is just ongoing staffing and what it looks like in [community organisation] and how it fits into everything that we’re doing here. Obviously just with the time lots of things have already changed in terms of who’s working on the project.”* P1
	Engagement	*“There are some people now who are wanting things face-to-face. So that can be a barrier. And also having access to online. If you looking at it from a cultural perspective, not everyone’s got, [from] a socioeconomic perspective, not everyone’s got Internet…”* P15
Adoption facilitator	Engagement	*“We’ve got a good IT [Information Technology] team here. We have the room set up. We’ve got great facilitators. They’ve got the will. Everyone’s excited about it. I think it’s just so wonderful to actually support, financially support a research team and then being able to implement a program once that research is finished.”* P10
	Knowledge	*“I really enjoy learning by watching and observing other people and thinking back to how I can use that in my own practice. So, I think I’m really grateful that I will be doing the first group at least, and - if not the second as well, with [Co-Facilitator Name], and he’s experienced in delivering online groups.”* P8
Appropriateness facilitator	Reach	*“I already have rapport with the young people that will be joining the program. So I think that that will really help for both their comfort sake, but also for content to be discussed. I think that would be helpful”* P14

**Table 3:** Outcomes and categories from after the implementation (T1)

T1 outcomes	Categories	Exemplar quotes
Acceptability barrier	Progress	“*I think it was a fairly slow start in terms of implementing the program, getting that underway”* P4
Acceptability facilitator	Relationship	*“I suppose the support from the university has been very consistent and very available. So I think having those regular meetings and the systems that we set up with the sharing of documentation was very enabling of the recruitment process. I think that worked well.”* P11
	Support	*“I’m used to seeing training platforms where you get given a video to watch, so it was really nice not having that and having someone to interact with, and I think it was really helpful [Researcher Name] gave us time and space to ask questions as she was going, rather than wait until the end, that kind of lecture style.”* P14
Adoption barrier	Structure	*“I think maybe the six weeks was a little bit too long, definitely – I think it was week five for us or week four… that we only had two [participants] to attend.”* P16
	Reach	*“I think the only other thing that it only really is a small group of people that we have were available to participate in the program….the number that fit the eligibility criteria is actually quite a lot smaller, and then the people who can do the dates that are available obviously get quite small.”* P1
Adoption facilitator	Knowledge	*“I guess because I had had colleagues who had run it before, that helped me in terms of facilitating it, because I was able to speak to [two group co-facilitators/leaders] and say, how did that work? For you, how did this session work? What were some of the things?”* P7
Appropriateness facilitator	Goal	*“I think we’re really interested in the program and I think the way we facilitate groups works well with that program. As an organisation the fact that we already delivered online, well we had a format for doing that, I think that was really good, so it blends in really well with the structures that we already had going. It filled a gap in a service provision.”* P9
Sustainability Other	Support	*“At the moment, our direct service, our counselling program, direct service delivery staff have not been affected with those cuts. But just say a challenge would be that if there was a requirement for a reduction in counselling staff hours, there’s additional projects that would be affected rather than our support services delivery. So that could be a potential challenge. It’s not an existing one in reality at the moment. But should that happen, certainly that would be a challenge to being able to do the work.” P11*

## Results

### Anticipated potential barriers and facilitators before implementation (T0)

Exemplar quotes from T0 are presented in Table 2. One participant could not identify any potential barriers at T0, while the remaining interviewees named both facilitators and barriers to the upcoming delivery of Recapture Life. The anticipated barriers at T0 were resistance from staff and participants, recruitment problems, different target groups, rules and regulations associated with research studies, collaboration between the community organisations and the university, slow implementation pace, a higher administrative burden, time needed to get familiar with the content, technical and internet requirements, and staff turnover. One participant commented that a challenge was that the program was new both in terms of content and delivery mode:

*I think perhaps maybe not ‘barriers’, but challenges might be just my unfamiliarity with the content and just that being a challenge to overcome on a week-by-week basis. I think the technology will be interesting as well. We can factor for all sorts of different things, make sure things are working on our end. But at the end of the day, glitches happen. The manner of overcoming that. Also, as I mentioned before, I’ve not run a like a larger group on a video conferencing format, so that’ll be new. So that’ll be something to overcome.* P14

The anticipated facilitators were support from the organisations’ management, the organisations’ good reputation amongst survivors, the training provided by the university, the communication between the university and the organisations, the flexibility of the program and manual, and that two staff members would be delivering the intervention.

### Barriers and facilitators experienced during implementation

Exemplar quotes from T1 are presented in Table 3. Most of the potential barriers and facilitators mentioned at T0 were confirmed at T1, but not all of them. Staff turnover was predicted as a potential barrier at T0 and was mentioned again at T1, and the same was true for recruitment problems. Similarly, the fit within the community organisation and the chance to provide peer support were mentioned as facilitators at both timepoints, just like the support and training provided by the university and the communication between the academic research team and the community organisation. One difference between T0 and T1 was that participants expected AYAs to prefer face-to-face delivery and that there would be problems with internet access and stability, both being possible barriers. However, this was not mentioned as a barrier at T1. Instead, it was the staff who preferred face-to-face delivery and were struggling with the online mode during the implementation.

*I think it’s easier face-to-face and I was struggling with the format. I think we’ll get better at doing the online delivery and that certainly changed quite rapidly once we started going, because it was a new platform for us at that time.* P9

*Then the second challenge I suppose, was just that online space and, we were already getting used to it because of COVID, so we had a bit of practice in that sense of getting used to it, but it’s still a very foreign platform for me to use as a workshop or – I’m so used to doing that face-to-face [in person] rather than with a screen, so that was a challenge.* P14

While the structure with two staff member per group was a facilitator at both timepoints, disagreements between them was mentioned as an experienced barrier at T1.

*I felt that our two different ways of being, our two different therapeutic ways, for me, was a bit more of a barrier.* P8

Relationships were the overall most frequently mentioned barrier and facilitator, across both timepoints. This included the relationship between the university and the community organisation, the relationship between management and staff within the organisation, and the relationship between the community organisation and the survivors.

### Categories and outcomes

**Fig. 2:**
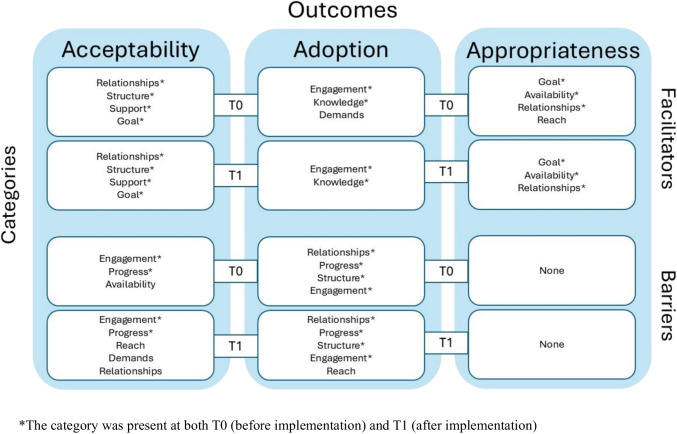
Outcomes and categories mapped onto the Proctor framework

The categories and the outcomes for T0 and T1 are both shown in Fig. 2. At T0, nine subcategories were identified: engagement, reach, relationships, progress, demands, support, structure, goal, and knowledge. These corresponded to three different areas from the Proctor framework: acceptability, adoption, and appropriateness. The subcategory *engagement* covered resistance (from both participants and staff) as a barrier, as well as the community organisation’s keenness to implement Recapture Life as a facilitator. *Reach* included recruitment problems as a barrier and that community organisations targeted different age groups as a facilitator. *Relationships* encompassed the collaboration between the university and the community organisation as both a barrier and facilitator and the community organisation’s reputation as a facilitator. *Progress* only included barriers, in the form of slow implementation pace, research administration, and staff turnover. *Demands* also included barriers, such as unclear rules and regulations, computer and internet needs, and that the staff had to get familiar with the Recapture Life content. *Support* included facilitators such as resources within the community organisation, the training provided by the university, and communication between the university and the community organisation. *Structure* covered the properties of Recapture Life and its manual and flexibility, and that there were several therapists as facilitators, but also the online delivery as a barrier. *Goal* included facilitators such as being able to provide peer support and that the implementation would benefit the community organisation. *Knowledge* was both a barrier, as it was a new method, and a facilitator, referring to the staff’s previous experience and skills, and the opportunity to learn from experience. Knowledge did not only cover the content but also the method of delivery.

The nine subcategories from T0 were also present at T1, with three additional subcategories being identified: availability, alterations, and contextual factors related to the COVID-19 pandemic. *Availability* covered scheduling (making it possible for AYAs to participate at a certain time of the day), as well as timing (which month the implementation started). *Alterations* included both adaptations of and deviations from the program. These were neither barriers nor facilitators but were mentioned as part of the last question of the interview (other comments). This also included recommended modifications necessary for sustainability, the fourth outcome from the Proctor framework.

The majority of the T1 interviews were conducted after or during the Australian lockdown (which started in March 2020 and ended October 2021). The *COVID-19* pandemic was considered both a barrier and a facilitator to the program’s implementation. As a category, it did not fit neatly into the Proctor framework but rather crosscut all outcomes. As a facilitator, COVID-19 created circumstances that primed AYAs to be especially open to telehealth support:

*I guess it’s hard to answer because I don’t – it’s never been rolled out when COVID wasn’t around so I’ve got no comparison, but I think actually COVID helped because [the participants] were all at home and were all looking for something to do. So, Recapture [Life] came at quite a good time because it was a group that got it and understood, whereas real-life groups weren’t happening.* P9

Yet, the physical separation of staff (caused by the lockdown) was also mentioned as a barrier belonging to the subcategory relationships. Relationships further included a new barrier at T1 in the form of the intervention groups being too diverse, which affected group dynamics.

## Discussion

To respond to the psychological needs of AYA cancer survivors, we developed Recapture Life—an online intervention to teach adaptive coping skills to promote resilience in early survivorship [8]. The current study aimed to evaluate the process of implementing Recapture Life in community settings, focusing on facilitators and barriers. The participants were psychosocial healthcare providers with prior experience in CBT and were also provided with training in delivering the intervention. They were interviewed after training (but before delivering the intervention) and after delivery. The findings diverged between the two timepoints regarding different aspects. One was the emergence of new categories after the delivery and the second that an anticipated barrier for the AYAs turned out to be a barrier for the facilitators instead.

We identified 12 subcategories at T1 (after intervention delivery) which could be mapped onto four different outcomes from the Proctor framework. These were engagement, reach, relationships, progress, demands, support, structure, goal, knowledge, availability, alterations, and COVID. The first nine of these were also mentioned at T0 (before intervention delivery). While the staff members’ anticipations and predictions were speculative at T =, most of them were confirmed at T1, after delivery of the intervention. The overall most frequently mentioned barrier and facilitator, across both interviews, was relationships. As a facilitator, the subcategory relationships represented the good relationship the community organisations had with the AYAs and their reputation. The relationship between the community organisations and the university was also an important part of this. Because the university and community organisations had collaborated previously, staff entered the project with well-established channels of communication and a sense of mutual trust, which supported implementation. However, within the context of the formalised research collaboration, different procedural responsibilities and administrative requirements required of both community organisations and the university, respectively, sometimes created delays or added complexity to the implementation process. The relationship between staff was an expected facilitator before delivery, but different approaches sometimes caused frustration instead, meaning this was both a facilitator and barrier after the implementation.

The staff members in our implementation study received extensive training and support. Both the training (delivered by the collaborating university) and the support from the community organisation were seen as important facilitators in this study, as well as the communication between the university and the community organisation/s, at both timepoints. The importance of the support seen in our study echoes other studies. A study investigating Australian health professionals’ perceptions of the feasibility and acceptability of a community-based model of psycho-oncology care revealed that most clinicians were willing to adopt the changes into practice, with one of the main facilitators being appropriate support and education for community-based clinicians [24]. Psycho-oncology implementation challenges are multifaceted and occur across various domains, including challenges associated with changing processes of clinical care, clinician knowledge, and the culture within an organisation [20]. We did not explicitly investigate the changing process, but both clinician knowledge (the skill and expertise of the staff) and the culture within the organisation were also labelled as facilitators at both timepoints. This is in line with an American study, reporting that most the barriers to implementing psycho-oncology evidence-based interventions into routine practice are encountered at the organisational or institutional level [23].

One category considered both a barrier and a facilitator was the COVID-19 pandemic. Early in the pandemic, Australia implemented a comprehensive set of measures to support telehealth [31–33]. This shift was prioritised for Australian cancer care to both protect vulnerable individuals and reduce rural–urban disparities [34]. While some of the interviews, especially for T0, were conducted before the onset of the COVID-19 pandemic, the majority of the T1 interviews were conducted after or during the Australian lockdown. Before the delivery, some participants expressed concern that the AYAs would prefer a face-to-face intervention and that unreliable/unstable Internet access could be a barrier. After the delivery, this was not mentioned as a concern for the AYAs, but it was mentioned as limiting for the staff. This may reflect clinicians/staff members’ own hesitations about the delivery modality [35]. Due to many AYAs still attending school or university, where lessons and lectures were delivered online during the lockdown, they might have had more experience with the online mode than the staff. However, it is also possible that the survivors were already used to receiving telehealth cancer care due to the Australian shift in policy measures. A recent American study on the transition from in-person to virtual delivery of community support for adults with cancer reported that younger adults were more likely to endorse the transition to online platforms [36]. It is therefore also possible that age was a contributing factor, as the staff, on average, were 23 years older than the AYAs.

A systematic review of staff-reported barriers and facilitators to implementation processes reported that they should be assessed across three different domains: system, staff, and intervention [37]. These domains were present in our material (with the community organisation representing ‘the system’) and were mentioned frequently at both timepoints. However, we also report that recruitment of AYAs was a major barrier to implementation, with participants speculating that this may be due in part to a lack of interest or time amongst survivors, as well as to few people meeting the eligibility criteria and difficulties reaching the right audience. Previous research has shown that the key to recruiting cancer AYAs for research is having the right clinical staff approach them [38], so recruitment through community organisations might have contributed to the limited reach. At the same time, the participants stressed that the Recapture Life program was beneficial to the AYAs and that it filled a gap in the services provided by the community organisations. As there are few studies on CBT for AYAs with cancer [11] and community organisations at the same time move to virtual platforms [36], a key future direction for implementation research is to strengthen recruitment pathways while using rapid-cycle learning approaches to iteratively evaluate and refine implementation.

### Limitations

While 18 out of 19 community staff members agreed to participate before implementation, only 9 were available again after implementation due to staff turnover. Another limitation was the slow pace of implementation. This meant that there was a lag between interviews, which increased the risk for staff leaving the community organisation. Both staff turnover and the slow implementation pace were predicted as potential barriers at T0 and mentioned again at T1. This means a risk of loss of continuity across timepoints and introduces the risk that individuals who voiced expectations, concerns, or predicted facilitators/barriers at T0 did not provide corresponding follow-up reflections at T1. Accordingly, we cannot be certain whether changes across timepoints reflect actual shifts in perception or simply differences between who contributed at each timepoint. 

## Conclusions

This study highlights the potential for psychological programs to be delivered via digital means by skilled and well-trained psychosocial staff in community and not-for-profit settings. As in academic settings, participant recruitment amongst AYAs remained a key challenge, and staff turnover emerged as an additional barrier in community settings. We identified several processes critical to supporting the success of intervention delivery, including offering comprehensive staff training, strong communication between academics and community partners, and fostering a culture of support within the organisation for the new program.

## Data Availability

The data that support the findings of this study are available from the corresponding author on reasonable request. The data are not publicly available due to privacy and ethical restrictions.
